# Identification of Novel Thermosensors in Gram-Positive Pathogens

**DOI:** 10.3389/fmolb.2020.592747

**Published:** 2020-11-26

**Authors:** Pilar Fernández, Alejandra Raquel Díaz, María Florencia Ré, Lucía Porrini, Diego de Mendoza, Daniela Albanesi, María Cecilia Mansilla

**Affiliations:** ^1^Instituto de Biología Molecular y Celular de Rosario (IBR-CONICET), Rosario, Argentina; ^2^Departamento de Biología, Bioquímica y Farmacia, Universidad Nacional del Sur, Centro de Recursos Naturales Renovables de la Zona Semi-árida (CERZOS-CONICET), Bahía Blanca, Argentina; ^3^Departamento de Microbiología, Facultad de Ciencias Bioquímicas y Farmacéuticas, Universidad Nacional de Rosario, Rosario, Argentina

**Keywords:** thermosensor, gram positive pathogen, ABC transporter, two component system, signalling

## Abstract

Temperature is a crucial variable that every living organism, from bacteria to humans, need to sense and respond to in order to adapt and survive. In particular, pathogenic bacteria exploit host-temperature sensing as a cue for triggering virulence gene expression. Here, we have identified and characterized two integral membrane thermosensor histidine kinases (HKs) from Gram-positive pathogens that exhibit high similarity to DesK, the extensively characterized cold sensor histidine kinase from *Bacillus subtilis*. Through *in vivo* experiments, we demonstrate that SA1313 from *Staphylococcus aureus* and BA5598 from *Bacillus anthracis*, which likely control the expression of putative ATP binding cassette (ABC) transporters, are regulated by environmental temperature. We show here that these HKs can phosphorylate the non-cognate response regulator DesR, partner of DesK, both *in vitro* and *in vivo*, inducing in *B. subtilis* the expression of the *des* gene upon a cold shock. In addition, we report the characterization of another DesK homolog from *B. subtilis*, YvfT, also closely associated to an ABC transporter. Although YvfT phosphorylates DesR *in vitro*, this sensor kinase can only induce *des* expression in *B. subtilis* when overexpressed together with its cognate response regulator YvfU. This finding evidences a physiological mechanism to avoid cross talk with DesK after a temperature downshift. Finally, we present data suggesting that the HKs studied in this work appear to monitor different ranges of membrane lipid properties variations to mount adaptive responses upon cooling. Overall, our findings point out that bacteria have evolved sophisticated mechanisms to assure specificity in the response to environmental stimuli. These findings pave the way to understand thermosensing mediated by membrane proteins that could have important roles upon host invasion by bacterial pathogens.

## Introduction

Organisms permanently sense and respond to environmental changes in order to adapt and survive. For this, bacteria employ a large number of two-component systems (TCS) consisting of pairs of sensor histidine kinases (HKs) and response regulators (RRs). TCSs perceive specific external signals including extreme pH, high salinity, antibiotics and temperature, among others, and usually give a transcriptional response to the initial stimulus. Up to date, only few TCS responsible for low temperature perception in different organisms have been characterized (Jin et al., 1993; Suzuki et al., 2000; Braun et al., 2008; Chakraborty et al., 2010; Steele et al., 2012; Najnin et al., 2016). For more than 20 years, our group has been studying the DesK/DesR TCS responsible for cold adaptation in the Gram-positive model bacterium *Bacillus subtilis* (for a review see de Mendoza, 2014), turning it into a paradigmatic transduction pathway. This TCS is conserved among Firmicutes, including Gram-positive pathogens, where temperature adaptive strategies play a significant role. Although the thermosensing ability of these paralog proteins has not yet been addressed, they emerge as promising candidates to accomplish this function.

The HK DesK lacks an extracellular domain and works as a homodimer, with each protomer contributing with five transmembrane (TM) helices to the sensory domain. It perceives changes in cytoplasmic membrane properties associated to a temperature downshift, like fluidity and thickness (Cybulski et al., 2002, 2010; Porrini et al., 2014; Saita et al., 2015). Upon a cold shock, DesK adopts a kinase-ON state phosphorylating DesR, its cognate RR. DesR-P is able to bind to the promoter region of the *des* gene and induces its expression (Aguilar et al., 2001; Cybulski et al., 2004). *des* encodes a Δ5-desaturase (Δ5-Des), which introduces a *cis* double bond into the acyl-chain of phospholipids increasing cytoplasmic membrane fluidity (Altabe et al., 2003). In the context of a fluid membrane, DesK adopts its phosphatase catalytic state, dephosphorylating DesR-P and turning off *des* expression (Albanesi et al., 2004). Crystal structures of the DesKC:DesR complex, trapped in the phosphatase and the phosphotransferase functional states allowed to uncover the structural bases of HK phosphatase/phosphotransferase control. The DesK:DesR structures revealed that both partners contribute residues to build the catalytic centers for TCS-mediated phosphotransfer and dephosphorylation reactions. Moreover, it was established that the HK is the component that drives the construction of a given active site by selecting a conformation of the RR that is prone for transfer reactions on its catalytic aspartate. The transition from the phosphatase to the phosphotransferase state of the HK, switches the distance and orientation of the catalytic histidine side chain with respect to the RR aspartate, allowing for the distinct reaction mechanisms to take place and ensuring the unidirectional flow of information (Trajtenberg et al., 2016).

**FIGURE 1:**
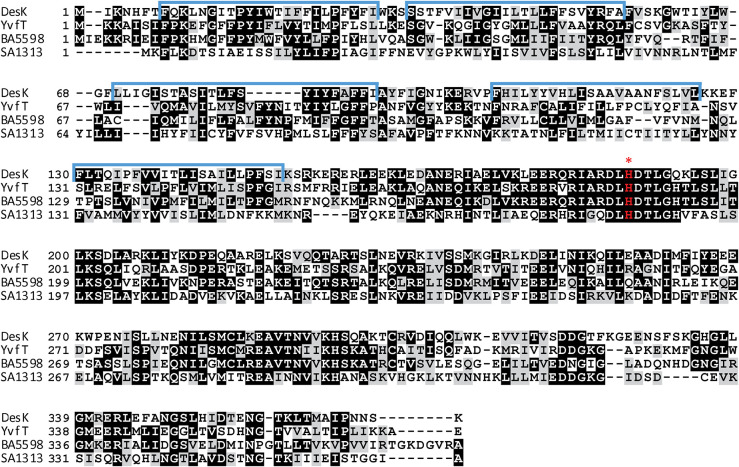
Amino acid sequence alignment of HKs, DesK, YvfT, BA5598 and SA1313. Identical amino acids are shown highlighted in black and similar ones are highlighted in gray. TMS are marked above the DesK sequence in blue (Cybulski et al., 2010). The red asterisk indicates the phosphorylatable His.

**FIGURE 2:**
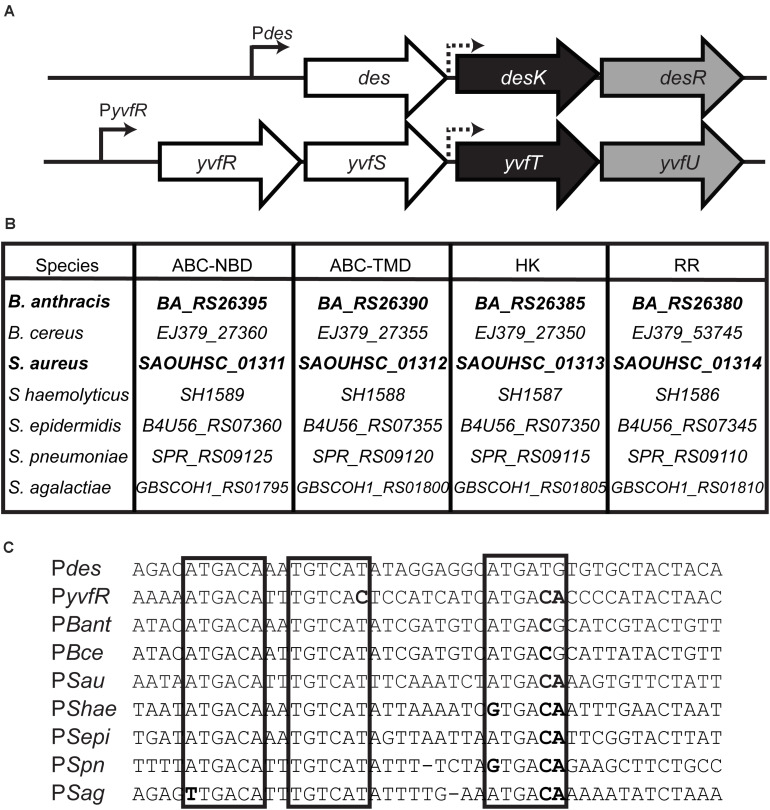
**(A)** Chromosomal organization of the *B. subtilis* homologous TCS regions. Sensor HK genes are shaded in black, RR in gray and upstream genes in white. *des*: desaturase; *yvfR* ABC transporter nucleotide binding domain (ABC-NBD), *yvfS* ABC transporter TM domain (ABC-TMD). Continuous arrows represent promoters and dashed arrows putative promoters. **(B)** Conserved clusters among Gram positive pathogens. The gene names of different species were extracted from GeneBank. *B. anthracis* str. Ames, *B. cereus* ATCC 14579, *Staphylococcus aureus* RN4220, *S. haemolyticus* JCSC1435, *S. epidermidis* 1457, *Streptococcus pneumonia* R6, *S. agalactiae* COH1. **(C)** Alignment of promoter regions. Direct and inverted repeats involved in RR binding in P*des* promoter (Najle et al., 2009) are marked in boxes. Sequence differences respect to P*des* are shown in bold.

*B. subtilis* contains a paralog of the DesK/DesR TCS of unknown function, named YvfT/YvfU. The amino acid identity between YvfT and DesK is 37% while the identity between YvfU and DesR is 58% (Figure 1 and Supplementary Figure S1). The *des* gene, target of DesK/DesR, is located right upstream of the *desK/desR* operon while upstream of *yvfT/yvfU*, there are two genes, *yvfR* and *yvfS*, coding for subunits of a putative ATP binding cassette (ABC) transporter (Figure 2A). YvfR is homologous to the nucleotide-binding domain of the transporter, while YvfS resembles the permease. The four-gene cluster, coding for the ABC transporter system and the TCS, is conserved among Firmicutes, including important human pathogens, like *Staphylococcus aureus*, *Staphylococcus haemolyticus*, *Staphylococcus epidermidis*, *Streptococcus pneumoniae*, *Streptococcus agalactiae*, *Bacillus cereus*, and *Bacillus anthracis* (Figure 2B). Interestingly, in spite of encoding proteins with complete different biological functions, the promoter regions of these ABC transporter coding genes and *des* are quite similar. All these promoters have three RR binding regions, with an inverted and a direct repeat similar to that described for the *des* promoter (P*des*) (Figure 2C; Najle et al., 2009).

In this work, we investigated the functionality of three TCS homologous to DesK/DesR: YvfT/YvfU from *B. subtilis*, BA5598/BA5597 from *B. anthracis* and SA1313/SA1314 from *S. aureus*. Using a *B. subtilis desK* knockout reporter strain, we demonstrated that the HKs YvfT, BA5598 and SA1313 are able to perceive a downshift in growth temperature. We also analyze the ability of these HKs to respond to changes in the cytoplasmic membrane physical properties, due to variations in lipid composition, at a constant temperature. We report that each HK responds to a particular set of stimuli with different sensitivity, probably because they belong to bacteria that inhabit different ecosystems. The *in vivo* approach shown here uncovered novel temperature responsive HKs found in Gram-positive pathogens.

## Materials and Methods

### Bacterial Strains and Growth Conditions

Bacterial strains and plasmids used in this study are listed in Table 1. *Escherichia coli* and *B. subtilis* strains were routinely grown in Lysogeny Broth (LB) at 37°C. When necessary, *B. subtilis* was propagated in Spizizen Salts (Spizizen, 1958), supplemented with 0.5% of glycerol, 0.01% tryptophan, 0.01% phenylalanine and trace elements (Harwood and Cutting, 1990). This medium was designated SMM. Isoleucine (0.01%) was added when indicated.

**TABLE 1:** Strains and plasmids used in this study.

Strain or plasmid	Relevant characteristics	Source
**Strains**		
RN4220	*S. aureus*	Laboratory stock
34F2	*B. anthracis* Sterne (pXO1^+^ pXO2^–^)	Laboratory stock
JH642	*B. subtilis trpC2 phe*A1	Laboratory stock
DAK3	JH642 *desKR::Kn* P*xylA-desR amyE::*P*des-lacZ*	Saita et al., 2015
CM21	JH642 *desKR::Kn thrC::*P*xylA-desR amyE::*P*des-lacZ*	Albanesi et al., 2004
CM28	JH642 *desKR*::*Kn amyE::*P*des-lacZ*	This work
**Plasmids**		
pHPKS	*B. subtilis* replicative vector of low copy number	Johansson and Hederstedt, 1999
pGES40	P*xylA* cloned into pBluescript SK(+) (Stratagene)	Diez et al., 2012
pARD7	pHPKS derivative with P*xylA* cloned in *Sac*I	Díaz et al., 2019
pLarC1	pHPKS derivative with P*xylA* cloned in *Hin*dIII and *Eco*RI sites	Trajtenberg et al., 2014
pLUP124	pHPKS-*PxylA-desK*	Porrini et al., 2014
pPF23	pHPKS *PxylA-yvfT*	This work
pLUP161	pHPKS *PxylA-yvfTU*	This work
pH5598	pHPKS *PxylA-BA_RS26385*	This work
pPxyl-HKsa	pHPKS-*PxylA-SAOUHSC_01313*	This work
pQE30-32	*E. coli* expression vector (fusion to His tag)	QIAGEN
pMAL-c2X	*E. coli* expression vector (fusion to MBP tag)	New England Biolabs
pDA327	pQE32*-desKC*	Albanesi et al., 2004
pPF46	pQE30-*yvfTC*	This work
pQE5598c	pQE32-*BA_RS26385C*	This work
pPF172	pQE30-*SAOUHSC_01313C*	This work
pPF177	pQE30-*SAOUHSC_01314*	This work
pPF28	pMAL-c2X-*yvfU*	This work
pM5597	pMAL-c2X-*BA_RS26380*	This work
pCMdesR	*desR* cloned into pETGEXCT	Albanesi et al., 2004

Antibiotics were used at the following final concentrations: lincomycin (Lm) 12.5 μg mL^–1^; erythromycin (Erm) 0.5 μg mL^–1^; chloramphenicol (Cm) 5 μg mL^–1^; kanamycin (Km) 5 μg mL^–1^; cerulenin (Cer) 2 μg mL^–1^; ampicillin (Amp) 100 μg mL^–1^.

For the experiments involving expression of the HKs under the control of the inducible P*xylA* promoter, xylose was added to the media as indicated in each case.

### Plasmids and Strain Construction

The genes encoding the different HKs and RRs were PCR-amplified using chromosomal DNA from *B. anthracis* 34F2, *S. aureus* RN4220 or *B. subtilis* JH642, and the corresponding oligonucleotides indicated in Table 2. In all cases Q5^®^ high-fidelity DNA polymerase (New England Biolabs) was used and the identity and correct sequence of the cloned fragments were corroborated by sequencing.

**TABLE 2:** Oligonucleotides used in this study.

Target gene	Name	Sequence
*yvfT*	yvfTBamUp	AGGATCCAAGAAGCGGTGTAATACATGA
	IBR	AATCGGTACCTGGATCCTCCAATCATTCTGC
*yvfTC*	yvfTCBamUp	GGATCCCGGAGGATTGAGCTTGAAG
	IBR	AATCGGTACCTGGATCCTCCAATCATTCTGC
*yvfU*	yvfUBamUp	GGATCCATTCGTCTGTTTATTGCTGAG
	yvfUDw	GGTCTGCAGTATATGGTTAGATCCAGC
*yvfT-yvfU*	yvfT-RBS	CCGTCGACAAGAAGCGGTGTAATACATG
	yvfUDw	GGTCTGCAGTATATGGTTAGATCCAGC
*SAOUHSC_ 01313*	1313Up	TGGATCCGGGCGGAATAAAATATG
	1313Dw	GGCTGCAGTAATTAAAGATGTCATGC
*SAOUHSC_ 01313C*	1313CUp	GGATCCGAATATCAAAAAGAAATAGC
	1313Dw	GGCTGCAGTAATTAAAGATGTCATGC
*SAOUHSC_ 01314*	SA1314ATG UP	ATGGATCCACATCTTTAATTATTGCAG
	SA59 DW	TCTGCAGTTTGTATTTAGATCCAGCC
*BA_RS26385*	Ba2552	AATCGATAGGGAGGCGTATAAATGATAGA GAAGAAG
	BAC3	AGGTCGACTTCGAATCATGCCCTCACC
*BA_RS26385C*	BAC5	CGGGATCCGTATGCGCAACTTTAATCAG
	BAC3	AGGTCGACTTCGAATCATGCCCTCACC
*BA_RS26380*	5597Up	TCGGGATCCGAAAACCTGTATTTTCAGGA TGATTCGAATTATTATTGC
	5597BxDw	AGGGGATCCTCTAGATCATATCCATCCCT TTTCTTC

*Restriction sites are underlined.*

*BA_RS26385* was first cloned into the *Cla*I and *Sal*I sites of pGES40, downstream of *xylR-*P*xylA* (Albanesi et al., 2004). The resulting plasmid was then digested with *Bam*HI and *Sal*I to release the fragment containing *xylR-PxylA-BA_RS26385* which was cloned into pHPKS digested with the same enzymes, obtaining plasmid pH5598. In this work we refer to the product of *BA_RS26385* gene, as BA5598 (previous gene name).

*SAOUHSC_01313*, was cloned into the *Bam*HI and *Pst*I of pARD7 (Table 1). The resulting plasmid was called pPxyl-HKsa. In this work we refer to the product of *SAOUHSC_01313* as SA1313.

*yvfT-yvfU* TCS was first cloned into pCR-bluntII-TOPO, then digested with *Sal*I and the resulting 5′-overhang filled with DNA polymerase I, Klenow fragment (Thermo Fisher Scientific). This construction was digested with *Spe*I, a restriction site contained in the vector, and the resulting fragment containing *yvfT-yvfU* was cloned into the *Sma*I and *Spe*I sites of pLarC1 (Table 1), yielding pLUP161. *yvfT* was amplified and cloned into pCR-bluntII-TOPO. The resulting plasmid was digested with *Spe*I and *Xba*I enzymes, and the 1.1 Kb fragment was cloned into pLarC1 digested with the same enzymes, yelding pPF23.

For overexpression in *E. coli*, the ORFs coding for the cytoplasmic domains of BA5598 (BA5598C), SA1313 (SA1313C), and YvfT (YvfTC), were amplified using the pair of oligonucleotides indicated in Table 2. The PCR fragments were purified and cloned into the *Bam*HI and *Pst*I sites of pQE30 or pQE32, yielding pQE5598c, pPF172, and pPF46 (Table 2).

To express the *B. anthracis* RR and YvfU as a maltose binding proteins (MBP) fusion protein to the N-terminus, the *BA_RS26380* gene, encoding BA5597 and *yvfU* were amplified and cloned into the *Bam*HI site of pMAL-c2X expression vector, to obtain pM5597 and pPF28, respectively.

To obtain the *S. aureus* RR with a His tag, the *SAOUHSC_01314*, encoding the RR SA1314, was cloned in *Bam*HI and *Pst*I sites of pQE30, yielding pPF177.

In all cases, amplicons were first cloned into pJet1.2 blunt (Invitrogen) or pCR-bluntII-TOPO, and sequenced.

### P*des-lacZ* Induction Analysis

*B. subtilis* DAK3, CM21, and CM28 strains (Table 1), all harboring P*des-lacZ* fusions, were transformed with the plasmids expressing the different HKs under the inducible *PxylA* promoter and, in parallel, with pARD7 (pHPKS-*PxylA*) to be used as a control. Strains were streaked on LB agar plates supplemented with 0.05% xylose and 60 μg mL^–1^ X-gal (5-bromo-4-chloro-3-indolyl-β-D-galactopyranoside) by duplicate. Plates were incubated for 5 h at 37°C and then one plate was transferred to 25°C overnight.

### Protein Overexpression and Purification

YvfTC, SA1313C, BA5598C, DesKC, and the RR SA1314 were expressed in *E. coli* M15/pREP4. Cells expressing the His-tagged recombinant proteins were grown in LB at 37°C with agitation to DO_600_ = 0.5. Then the culture was shifted to 20°C, and protein expression was induced by adding 0.3 mM isopropyl-β-D-1-thiogalactopyranoside (IPTG). After 16 h, cells were harvested, resuspended in lysis buffer [50 mM Tris–HCl pH 8, 500 mM NaCl and 1 mM phenylmethylsulfonyl fluoride (PMSF)] and treated with 1 mg mL^–1^ lysozyme for 30 min at 37°C. Subsequently, 5 μg ml^–1^ DNase plus 5 mM MgCl_2_ were added and after 20 min of incubation at room temperature cells were disrupted with an Emulsiflex^TM^ C3 homogenizer (AVESTIN). Cell debris were separated by centrifugation at 12000 × *g* for 20 min. The supernatant was recovered and incubated with pre-equilibrated Ni^+^-Nitrilotriacetic acid agarose resin (Qiagen), in the presence of 10 mM imidazole, for 3 h. The His_6_-tagged proteins were eluted with increasing concentrations of imidazole (Supplementary Figure S2) and dialyzed against 50 mM Tris pH 8, 300 mM NaCl and 10% glycerol. Proteins were conserved at −80°C for further experiments. Coomassie-stained SDS–PAGE of purification steps or final stored protein fractions are shown in Supplementary Figure S2.

Maltose binding protein-YvfU and MBP-BA5597 were overexpressed in *E. coli* XL1Blue. Cells expressing the MBP-tagged recombinant proteins were grown in LB at 37°C to a DO_600_ = 0.5 and then shifted to 30°C. Protein expression was induced by adding 0.3 mM IPTG. After 4 h, cells were harvested, resuspended in lysis buffer (20 mM Tris–HCl pH 7.4, 200 mM NaCl and 1 mM PMSF) and treated with 1 mg mL^–1^ lysozyme for 30 min at 37°C. Subsequently, 5 μg ml^–1^ DNase plus 5 mM MgCl_2_ were added. After 20 min of incubation at 37°C cells were disrupted with an Emulsiflex^TM^ C3 homogenizer (AVESTIN). Cell debris were separated by centrifugation at 12000 × *g* for 20 min. The supernatant was recovered and incubated with pre-equilibrated Amylose resin (New England Biolabs) for 1 h. The MBP fusion proteins were eluted with 10 mM maltose and dialyzed against 50 mM Tris pH 8, 300 mM NaCl and 10% glycerol. Proteins were conserved at −80°C for further experiments. Coomassie-stained SDS–PAGE of purification steps or final stored protein fractions are shown in Supplementary Figure S2.

GST-DesR was purified as previously described (Albanesi et al., 2004). Briefly, BL21/λDE3 strain transformed with pCMdesR was grown for 1 h at 37°C in LB and then 0.1 mM IPTG was added. Growth was continued for 4 h, cells harvested at 4°C and washed with 1× phosphate saline buffer, PBS (0.14 M NaCl, 0.27 mM KCl, 10.1 mM Na_2_HPO_4_, 1.8 mM KH_2_PO_4_). Cells were disrupted by sonication, centrifuged and the supernatant incubated with 50% glutathione-agarose resin (SIGMA), pre-equilibrated with 1 × PBS. GST-DesR was eluted with 5 mM Glutathione.

### *In vitro* Phosphorylation Assays

For the autokinase assay, purified YvfTC-Hisx6, BA5598C-Hisx6, SA1313C-Hisx6 at a final concentration of 10 μM were incubated in R Buffer (50 mM Tris pH 8, 200 mM NaCl, 1 mM DTT, 20% glycerol, 50 mM KCl, 1 mM MgCl_2_) containing 25 μM ATP in the presence of 0.25 μCi of [γ-^32^P]ATP/μl at room temperature. Reactions were initiated by addition of the corresponding HK.

For the phosphotransfer assays, each HK (10 μM) was allowed to autophosphorylate for 30 min in 150 μl of R buffer at room temperature. An aliquot of 15 μl was withdrawn (considered as time zero) and then the phosphotransfer assay was initiated by adding a volume of the corresponding RR to reach an equimolar final concentration with the HK. In all cases, aliquots were withdrawn at various time points, mixed with 5× sodium dodecyl sulfate-polyacrylamide gel electrophoresis (SDS-PAGE) loading buffer plus 50 mM EDTA to stop the reaction and kept on ice until loading.

### Bioinformatics

Protein sequences were analyzed with the program BLASTP (Altschul et al., 1990). Sequence alignments were performed using T-Coffee (Notredame et al., 2000) and drawn using Boxshade^1^. Molecular modeling was carried out with Swiss-Model^2^ (Waterhouse et al., 2018) and analyzed with PyMOL (PyMOL Molecular Graphic system, 2.0, Schrodinger, LLC, available at https://pymol.org/).

## Results

### *In vitro* Characterization of the DesK/DesR TCS Homologs

DesK is a multifunctional enzyme with autokinase, DesR phosphotransfer and phospho-DesR phosphatase activities that can assume different signaling states depending on the input signal. In the absence of the TM sensor domain, its cytosolic domain (DesKC) exhibits the three activities *in vitro* at 25°C (Albanesi et al., 2004). According to the two-component signal transduction paradigm, the HKs would first undergo autophosphorylation in a conserved histidine residue and then the phosphate would be transferred to the conserved aspartate residue in the receiver (REC) domain of its cognate transcriptional regulator. His188 has been identified unequivocally as the autophosphorylation site in DesK. A His188Val mutant was unable to receive the phosphate *in vitro* and to induce *des* transcription after a cold shock *in vivo* (Albanesi et al., 2004). His188 is located in a conserved domain called H-box, included in the dimerization and histidine phosphotransfer (DHp) domain, that defines the active site of phosphorylation (Trajtenberg et al., 2010). The alignment in Figure 1 clearly shows that the HKs under study contain this H-box, conserved in the HisKA_3 subfamily.

**FIGURE 3:**
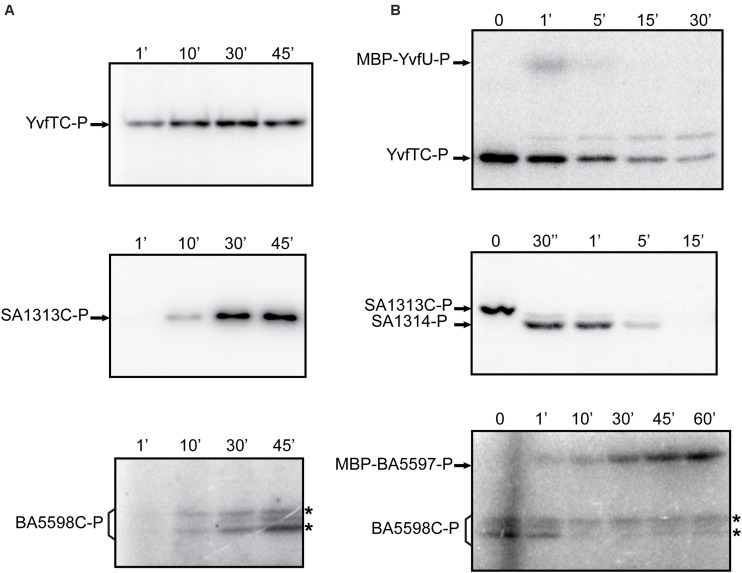
**(A)** Autophosphorylation assay of YvfTC, BA5598C, and SA1313C. The purified cytosolic domains were incubated with R buffer at room temperature. Reaction was initiated by the addition of [γ−^32^P] ATP. In the case of BA5598C, western blot analysis with anti-DesKC polyclonal antibodies showed that both bands marked with * correspond to the protein, while anti-His antibodies indicated that only the upper one possesses the 6xHis-tag (data not shown) **(B)** HKs phosphotransfer assay to cognate RRs. After 30 min of autophosphorylation at room temperature, each HK was mixed with an equimolar amount of its each cognate RR. After SDS-PAGE, gels were dried, exposed to a radioactive storage screen and analyzed using an Amersham Biosciences Molecular Dynamics Typhoon^TM^ FLA 7000 scanner. Pictures are representative of at least three independent experiments.

To analyze the catalytic activities of YvfT, BA5598 and SA1313 HKs, we expressed and purified the corresponding cytoplasmic soluble domains, YvfTC, BA5598C, and SA1313C, from *E. coli* (see section “Materials and Methods”). In all cases, the recombinant proteins included the complete DHp domain, containing the phospho-receiving His (His189, His187, and His185, respectively) and the ATP-binding domains (ABDs), since autophosphorylation involve specific contacts between these interacting domains (Trajtenberg et al., 2010). As shown in Figure 3A, YvfTC, BA5598C, and SA1313C are able to autophosphorylate in the presence of [γ-^32^P] ATP. YvfTC-P can be detected after 1 min of reaction, while detection of SA1313C-P and BA5598C-P requires longer incubation times. In all cases, the level of phosphorylation increased with time, reaching a plateau at 30 min for YvfTC and SA1313C (Supplementary Figure S3A).

To study the activation by phosphorylation of the RRs by their cognate kinases we produced the recombinant proteins in *E. coli*. As YvfU and BA5597 have similar molecular weight to the cytosolic domains of their corresponding HKs, YvfTC, and BA5598C, respectively, these RRs were fused to the MBP to be able to distinguish both partners in SDS-PAGE runs. As previously described, there is no detrimental effect of GST or MBP tags on RR recognition and phosphorylation (Albanesi et al., 2004; Tsai, unpublished). Besides, substrate specificity in HK-RR pairs is really high. Cognate pairs can identify each other in bacterial cells, where up to 300 HK-RR pairs are present. Such specificity is determined by a “specificity code” present in the contact surfaces: DHp in HKs and REC domain in RR (Skerker et al., 2008; Capra et al., 2012; Buschiazzo and Trajtenberg, 2019). The interacting surface between DesK and DesR in the phosphotransfer and dephosphorylation reactions has been described, and concerning DesR mainly involves its α1-helix and the β5α5 loop, with a few more contacts from the N-terminus of α5 and the beginning of the β4α4 loop. All these regions are conserved in the RRs under study in this manuscript (Supplementary Figure S1).

YvfTC, BA5598C, and SA1313C were allowed to autophosphorylate for 30 min in R buffer and then an equimolar amount of the cognate RR was added, promoting their phosphorylation (Figure 3B). For the YvfTC/MBP-YvfU pair, the level of RR-P was maximum after 1 min of reaction and then the radioactive label from both proteins decreased. After 15 min of incubation, MBP-YvfU-P could not be longer detected, while YvfTC-P could still be observed after 30 min of reaction (Figure 3B and Supplementary Figure S3B). Phosphotransfer from SA1313C-P to SA1314 was fast and very efficient. SA1313C-P could not be detected after 30 s of reaction while the level of SA1314-P reached a maximum within this incubation interval. Then, the level of the phosphorylated regulator decreased and was negligible after 15 min of reaction (Figure 3B and Supplementary Figure S3B). Finally, the phosphotransfer from BA5598C-P to MBP-BA5597 was slower and a longer time was necessary to observe a full transfer of the radioactive label. The level of MBP-BA5597-P increased during 60 min of incubation and no decay of the label was detected in these assay conditions (Figure 3B and Supplementary Figure S3B). To verify that the decrease of the level of YvfTC-P and SA1313C-P was due to specific phosphotransfer to MPB-YvfU and SA1314, respectively, the HKs were allowed to autophosphorylate for 30 min in R buffer and then incubated in the absence of their cognate RR, in the same reaction conditions. As shown in Supplementary Figure S5, YvfTC and SA1313C remained phosphorylated even after 45 min of incubation, confirming that the phosphorylated HKs are stable and do not lose the radioactive label spontaneously.

### Cross-Phosphorylation Between the Different HKs and DesR *in vitro*

**FIGURE 4:**
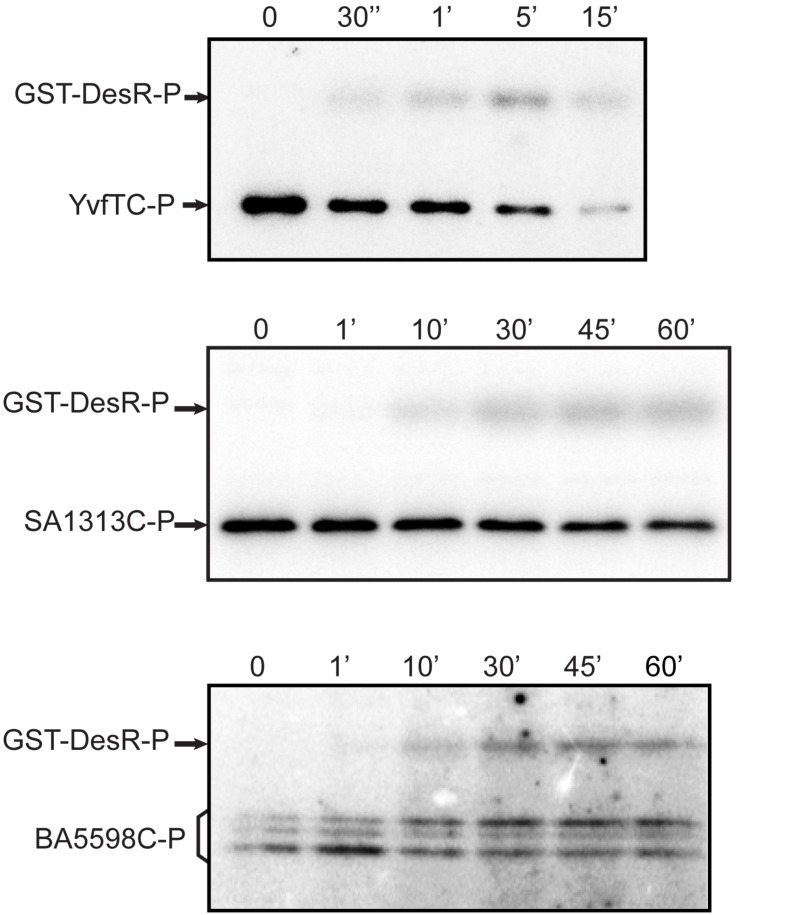
HKs phosphotransfer assay to DesR. After 30 min of autophosphorylation at room temperature, HKs were mixed with an equimolar amount of DesR. In all cases aliquots were taken at the indicated time points. Reactions were stopped by the addition of 5× sample buffer and loaded in 12% polyacrylamide gels. After SDS-PAGE, gels were dried, exposed to a radioactive storage screen and analyzed using an Amersham Biosciences Molecular Dynamics Typhoon^TM^ FLA 7000 scanner. Pictures are representative of at least three independent experiments.

Due to the high sequence similarity of the RRs (Supplementary Figure S1), we decided to investigate if YvfTC, SA1313C, and BA5598C are capable of recognizing and phosphorylating the non-cognate RR DesR. To this end, YvfTC, SA1313C, and BA5598C were allowed to autophosphorylate for 30 min and then an equimolar amount of purified GST-DesR was added. This tagged DesR version has been used successfully to test DesKC phosphotransfer and phosphatase activities (Albanesi et al., 2004). As shown in Figure 4, the phosphotransfer to GST-DesR was detected in all cases. GST-DesR was quickly phosphorylated upon incubation with YvfTC-P, the label on the RR could be detected after 30 s reaching a maximum after 5 min of reaction. Afterward, the level of GST-DesR-P and YvfTC-P decreased, being very low after 15 min of incubation (Figure 4 and Supplementary Figure S4). SA1313C-P and BA5598C-P were less efficient to catalyze the phosphotransfer to DesR and significant levels of GST-DesR-P could be detected only after 10 min of reaction. In both cases, the amount of GST-DesR-P remained almost constant after reaching the maximum, showing that dephosphorylation of the RR by the non-cognate HK was rather inefficient (Figure 4 and Supplementary Figure S4).

### *In vivo* Analysis of HKs Temperature Sensing Capacity

**FIGURE 5:**
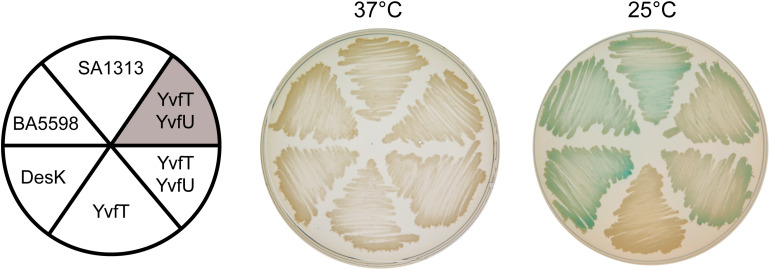
Complementation test of *desK* or *desK/desR* null mutants. CM21 strain (*desK:Kn thrC:*P*xylA-desR* P*des-lacZ*, white background) was transformed with plasmids expressing DesK, BA5598, SA1313, YvfT, or YvfT/YvfU under a xylose inducible promoter. CM28 (*desKR:Kn* P*des-lacZ*, shaded background) was transformed with a plasmid expressing YvfT/YvfU under a xylose inducible promoter. All transformants were grown in LB plates containing X-gal 60 μg mL^–1^ and xylose 0.05%. One plate was incubated at 37°C and the other was kept at 37°C for 5 h and then transferred to 25°C for 24 h.

The TM domain of DesK is essential for signal detection and modulation of its catalytic activities (Albanesi et al., 2004; Martín et al., 2009; Cybulski et al., 2010). Although the TM helices of DesK, YvfT, SA1313 and BA5598 have low amino acid sequence similarity, they share the same architecture (Figure 1). Thus, we decided to investigate whether YvfT, SA1313 and BA5598 are also able to detect temperature changes. Taking advantage that these HKs recognize and phosphorylate DesR *in vitro* (Figure 3), we analyzed their functional properties in a *B. subtilis* host. With this aim, we engineered a DesK-less host containing the β-galactosidase reporter gene fused to the desaturase promoter (P*des-lacZ*). In this strain, (CM21, Table 1), the levels of β-galactosidase activities rely on activation of P*des*, that ultimately depends on the flux of phosphate from the HK to DesR (Albanesi et al., 2004). The induction of transcription of the reporter gene by the HKs expressed in this strain can be easily monitored on solid media containing X-gal (5-bromo-4-chloro-3-indolyl-β-D-galactopyranoside), where the colonies turn blue if the HK is in the kinase-ON state and remain clear if the HK is in a kinase-OFF state. As shown in Figure 5, expression of BA5598 and SA1313 from a xylose-inducible promoter (P*xyl*) in CM21 rendered clear colonies at 37°C and blue colonies at 25°C. These results show that, similarly to DesK, both HKs specifically adopt a kinase-ON state at low temperature and phosphorylate DesR, stimulating transcription from P*des*. *In vivo* phosphorylation of DesR by the non-cognate HKs agrees with the *in vitro* experiments. Nevertheless, expression of YvfT in CM21 produced clear colonies both, at 37 and 25°C (Figure 5). This result suggests that this HK does not perceive low temperature stimulus, or alternatively, a mechanism to avoid cross talk between YvfT/YvfU and DesK/DesR systems could explain this observation. To test this possibility, we decided to co-overexpress YvfT together with YvfU from P*xyl* in either CM21 or in a *B. subtilis desKR* knock-out mutant strain harboring the P*des-lacZ* reporter fusion (CM28, Table 1). In both cases, we observed blue colonies at 25°C and clear colonies at 37°C, indicating that YvfT is able to adopt a kinase-ON state after a temperature downshift phosphorylating YvfU to activate expression of P*des-lacZ* (Figure 5). It should be noted that expression from P*des* is stimulated by YvfT/YvfU only when this TCS is overexpressed (Figure 5). Indeed, P*des-lacZ* induction was not detected in a *desK^–^ or desKR^–^* strains that maintain their wild type single chromosomal copy of the *yvfTU* operon (Aguilar et al., 2001; Saita et al., 2015). These results directly prove that both signaling pathways are not redundantly regulating *des* transcription under physiological expression conditions.

### Isothermal Perception of Changes in the Physical State of the Membrane

Activation of SA1313, BA5598, and YvfT upon cooling suggests that these HKs could be sensing changes in membrane properties through their TM helices. Based in previous experiments with DesK (Cybulski et al., 2002; Porrini et al., 2014), we analyzed whether fluctuations in membrane fatty acid (FA) composition at a constant temperature affect the sensory function of these homologous HKs.

#### Changes in the Proportion of Iso- and Anteiso-Branched Chain FAs

**FIGURE 6:**
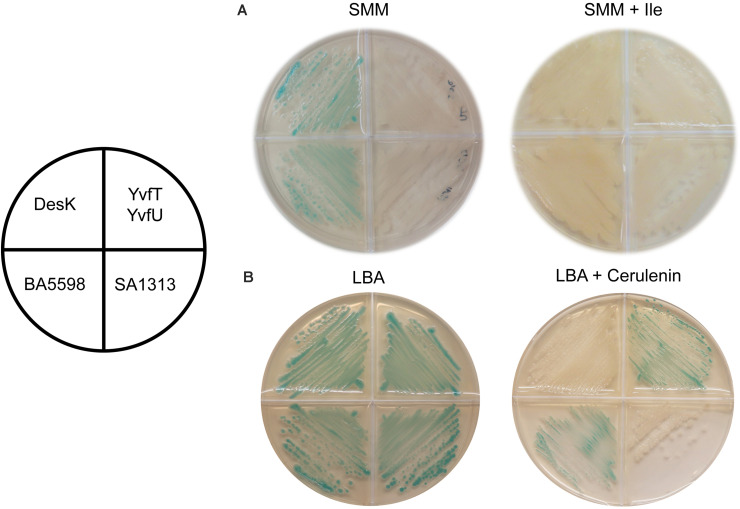
Response of homologous HKs to changes in membrane fatty acid composition. DAK3 strain (*desK:Kn* P*xylA-desR* P*des-lacZ*) transformed with plasmids expressing DesK, BA5598, SA1313, or YvfT/YvfU under a xylose inducible promoter were grown in SMM or LB plates containing X-gal 60 μg ml^–1^, xylose 0.05%, and isoleucine 0.01% or cerulenin 2 μg ml^–1^, as indicated. Plates were incubated at 37°C for 16 h **(A)**, or incubated at 37°C for 5 h and then transferred to 25°C for 24 h **(B)**.

*B. subtilis* adjust membrane fluidity at low growth temperatures by increasing the proportion of low melting point FAs, like unsaturated (UFAs) and branched chain fatty acids (BCFAs). The biosynthetic precursors of anteiso (ai)-BCFA are α-ketoacids derived from isoleucine (Ile), which is synthetized from threonine (Thr). If the culture media lacks Ile or Thr, JH642 strain cannot synthesize enough ai-BCFAs and the membrane becomes less fluid (Klein et al., 1999). Under such growth conditions, Δ5-Des expression is induced and UFAs are synthesized, even at 37°C, leading to membrane fluidity optimization. This induction is mediated by the DesK/DesR TCS demonstrating that DesK detects changes in the physical state of the cytoplasmic membrane rather than fluctuation in temperature to control *des* expression (Cybulski et al., 2002). Hence, we decided to test if the HKs homologous to DesK are also capable of perceiving isothermal membrane fluidity changes. To this end, we used a Thr prototroph *desK* knockout mutant strain, harboring the P*des-lacZ* reporter fusion, named DAK3 (Table 1). This strain was transformed with plasmids expressing the genes encoding the HKs SA1313 and BA5598 or the YvfT/YvfU TCS from a xylose inducible promoter. Transformants were grown in SMM agar plates containing X-gal and 0.05% xylose, in the presence or in the absence of isoleucine, at 37°C. As shown in Figure 6A, BA5598 was able to promote *lacZ* expression from P*des*, indicating that this *B. anthracis* HK is sensitive to the isothermal fluidity change. However, neither YvfT nor SA1313 were activated by isoleucine depletion (Figure 6A).

#### Effect of FA Chain Shortening by the Action of Cerulenin

We have proposed that membrane thickening is the main driving force for signal sensing by DesK (Cybulski et al., 2010; Martín and de Mendoza, 2013; Saita et al., 2015). A thicker membrane favors DesK kinase activity while its phosphatase state is promoted when membrane thickness is reduced. This regulation can be detected at a constant temperature *in vivo* using the antibiotic cerulenin. This compound is an inhibitor of the elongation of FAs and, in sub-inhibitory concentrations, favors the production and incorporation of short FAs into *B. subtilis* membrane phospholipids (Porrini et al., 2014). Lipids with shorter acyl chains generate thinner and less viscous membranes (de Mendoza, 2014). Thus, after cerulenin treatment of *B. subtilis* cells DesK adopts a kinase-OFF/phosphatase-ON state, even at low temperature, so transcription from P*des* is not induced at 25°C and hence, UFAs are not synthetized (Porrini et al., 2014). Therefore, we analyzed *in vivo* the response to low temperature of SA1313, BA5598 and YvfT in the presence of cerulenin. To this end, the reporter strain DAK3 expressing SA1313, BA5598 or YvfT/YvfU was grown in LB agar plates containing X-gal and xylose, supplemented or not with 2 μg/mL of cerulenin, at 25°C. As shown in Figure 6B, under these conditions, SA1313 does not promote P*des-lacZ* expression, indicating that, in the presence of cerulenin, it adopts a kinase-OFF conformation at low temperature. This result suggests that SA1313 senses the narrowing of the membrane caused by incorporation of shorter acyl chains. In contrast, BA5598 and YvfT exhibit a kinase-ON state, phosphorylating DesR or YvfU, respectively and inducing P*des-lacZ* transcription at 25°C, in spite of the presence of cerulenin. These results indicate that the decrease in membrane thickness that results from the treatment with cerulenin was insufficient to trigger the switch of these two HKs to the kinase-OFF state.

Altogether, these results demonstrate that, even though all the studied HKs have similar primary structure and respond to temperature changes, they differ in their sensitivity to changes in membrane physical properties. At a constant temperature, BA5598 can perceive fluidity changes promoted by a modification in the proportion of ai-BCFA while SA1313 can sense fluidity changes provoked by variation in the length of acyl-chains of membrane phospholipids. On the other hand, YvfT is insensitive to both stimuli.

## Discussion

Temperature is an important parameter that free-living organisms monitor constantly. In the case of bacterial pathogens, the expression of cold shock, heat shock and some virulence genes is coordinated in response to temperature changes. This behavior highlights temperature sensing as a key event for a fine-tuned orchestration of bacterial virulence and pathogenesis. TCSs are one of the mechanisms that bacteria use to sense and respond to variations in temperature, so understanding the stimulus perception of HKs and their transmission to the RRs would be a way to control their growth and spread.

In this work, we studied three HKs homologous to DesK: BA5598 from *B. anthracis*, SA1313 from *S. aureus* and YvfT from *B. subtilis*. Using *in vivo* assays, we demonstrated that these HKs are able to perceive a downshift in temperature, shifting its activity toward the kinase-ON state to promote transcription from P*des*, when expressed in *B. subtilis*. We found that BA5598 and SA1313 could recognize and phosphorylate the non-cognate RR DesR both *in vitro* and *in vivo*, while YvfT could only phosphorylate and activate DesR *in vitro*. We also determined that the co-overexpression of YvfT and YvfU in *B. subtilis* led to the induction of P*des-lacZ* expression upon a cold shock, without the assistance of DesR. This finding demonstrates that YvfU is capable of recognizing the operator sequences of the *des* promoter to activate transcription when temperature decreases. Under our experimental conditions, time course experiments demonstrated a similar phosphotransfer rate between YvfT-P and both MBP-YvfU or GST-DesR (Figures 3B, 4). However, in physiological conditions, YvfT or YvfT/YvfU do not cross-talk with the Des pathway since in *desK* or *desK/desR B. subtilis* null mutants the *des* gene is not turned on upon a cold shock (Aguilar et al., 2001; Saita et al., 2015). Interestingly, the molecular model of the YvfT:DesR complex shows that most of the molecular recognition determinants involved in cognate pair DesK:DesR recognition are present (Supplementary Figure S6). These results highlight that the cellular context is essential to avoid the cross talk between the YvfT/YvfU and DesK/DesR systems.

Furthermore, we determined that all the studied sensor proteins are activated by a temperature decrease, although they behave differently toward changes in the cytoplasmic membrane properties provoked under isothermal conditions. Changes in membrane fluidity caused by increasing the proportion of shorter FAs into phospholipids prompt DesK and SA1313 to adopt a phosphatase-ON state even at 25°C. However, this stimulus is not enough to turn off the kinase state of BA5598 and YvfT at this temperature. The motif conformed by three hydrophilic amino acids (Gln9, Lys10, and Asn12) located near the DesK N-terminal end of its first TM helix, called the sunken buoy, is critical to sense membrane thickness (Cybulski et al., 2010). At low temperatures, the membrane becomes thicker due to an increase in the lipid order, thereby trapping this hydrophilic motif inside the hydrophobic membrane environment, promoting the stretching of TM1 with the concomitant generation of the signal that finally promotes the kinase activity of DesK (Cybulski et al., 2010; Saita et al., 2015). In BA5598 and YvfT there is a Pro residue instead Gln9 in TM1, which could introduce a kink into this amphipathic domain changing the sensitivity of these HKs to membrane thickness variations.

Apart from temperature, BA5598 also responds to a decrease in membrane fluidity caused by the reduction of ai-BCFA in membrane phospholipids, adopting a kinase-ON state at 37°C. However, YvfT and SA1313 were unable to respond to this stimulus. Previous studies of P*des-lacZ* transcription at different temperatures, in the presence or absence of ai-BCFA precursors, revealed that each stimuli triggers a variation in membrane fluidity of a particular extent (Cybulski et al., 2002). Our results suggest that this set of HKs might have particular thresholds to acquire the kinase-ON state exhibiting different sensitivity to membrane fluidity changes.

In *toto*, we demonstrate here that YvfT, SA1313 and BA5598 respond to changes in environmental temperature adopting a kinase-ON state upon a cold shock, sensing different dynamic changes of membrane lipids. Finally, considering the similarity of the promoters of the putative ABC transporters and P*des*, as well as the high sequence identity between DesR and the studied RRs, we postulate that YvfT/YvfU, SA1313/SA1314, and BA5598/BA5597 control the expression of their cognate transporters. As the translocated molecules in each case are unknown, it is still unclear which is the response triggered by the cells upon a decrease in temperature and/or changes in the cytoplasmic membrane properties. Identifying which are the molecules exported by the ABC transporters would help to elucidate the function of these related TCSs and dissect their roles in bacterial pathogenesis.

## Data Availability Statement

The raw data supporting the conclusions of this article will be made available by the authors, without undue reservation.

## Author Contributions

PF, ARD, MFR, and LP performed the experiments. PF and MCM designed the study and conceived the experiments. PF, DA, and MCM wrote the manuscript, with input from ARD and DdM. All authors analyzed the data, have read, and approved the final version.

## Conflict of Interest

The authors declare that the research was conducted in the absence of any commercial or financial relationships that could be construed as a potential conflict of interest.

## Footnotes

1
https://embnet.vital-it.ch/software/BOX_form.html

2
https://swissmodel.expasy.org/
