# Corrigendum: Identification of novel thermosensors in gram-positive pathogens

**DOI:** 10.3389/fmolb.2022.1007054

**Published:** 2022-08-31

**Authors:** Pilar Fernández, Alejandra Raquel Díaz, María Florencia Ré, Lucía Porrini, Diego de Mendoza, Daniela Albanesi, María Cecilia Mansilla

**Affiliations:** ^1^ Instituto de Biología Molecular y Celular de Rosario (IBR-CONICET), Rosario, Argentina; ^2^ Departamento de Biología, Bioquímica y Farmacia, Universidad Nacional del Sur and Centro de Recursos Naturales Renovables de la Zona Semi-árida (CERZOS-CONICET), Bahía Blanca, Argentina; ^3^ Departamento de Microbiología Facultad de Ciencias Bioquímicas y Farmacéuticas, Universidad Nacional de Rosario, Rosario, Argentina

**Keywords:** thermosensor, gram positive pathogen, ABC transporter, two component system, signalling

In the published article, there was an error in Figure 1 as published. The blue line delimiting DesK’s first transmembrane segment should end at the aminoacid residue I30 instead of I24. The corrected Figure 1 and its caption:

**FIGURE 1:**
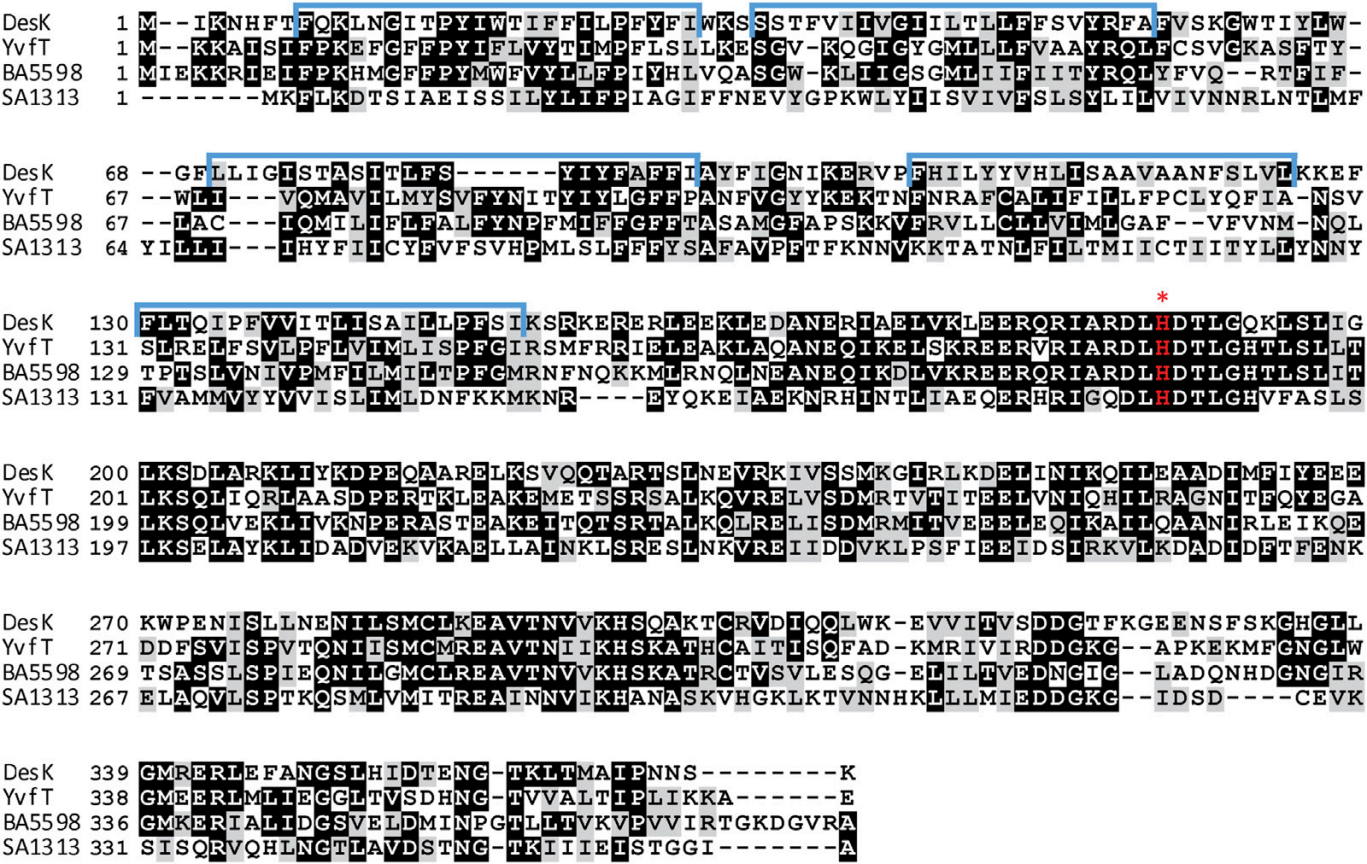
Amino acid sequence alignment of HKs, DesK, YvfT, BA5598 and SA1313. Identical amino acids are shown highlighted in black and similar ones are highlighted in gray. TMS are marked above the DesK sequence in blue (Cybulski et al., 2010). The red asterisk indicates the phosphorylatable His.

“Amino acid sequence alignment of HKs, DesK, YvfT, BA5598 and SA1313. Identical amino acids are shown highlighted in black and similar ones are highlighted in gray. TMS are marked above the DesK sequence in blue (Cybulski et al., 2010). The red asterisk indicates the phosphorylatable His)” appear below.

The authors apologize for this error and state that this does not change the scientific conclusions of the article in any way. The original article has been updated.

