# Supplementary material for: Identification of Novel Thermosensors in Gram-Positive Pathogens
Source: Front Mol Biosci. 2020 Nov 26;7:592747. doi: 10.3389/fmolb.2020.592747 (PMC7726353; doi:10.3389/fmolb.2020.592747)
Supplement: Supplementary file 1 [file Data_Sheet_1.PDF]

Supplementary Figures

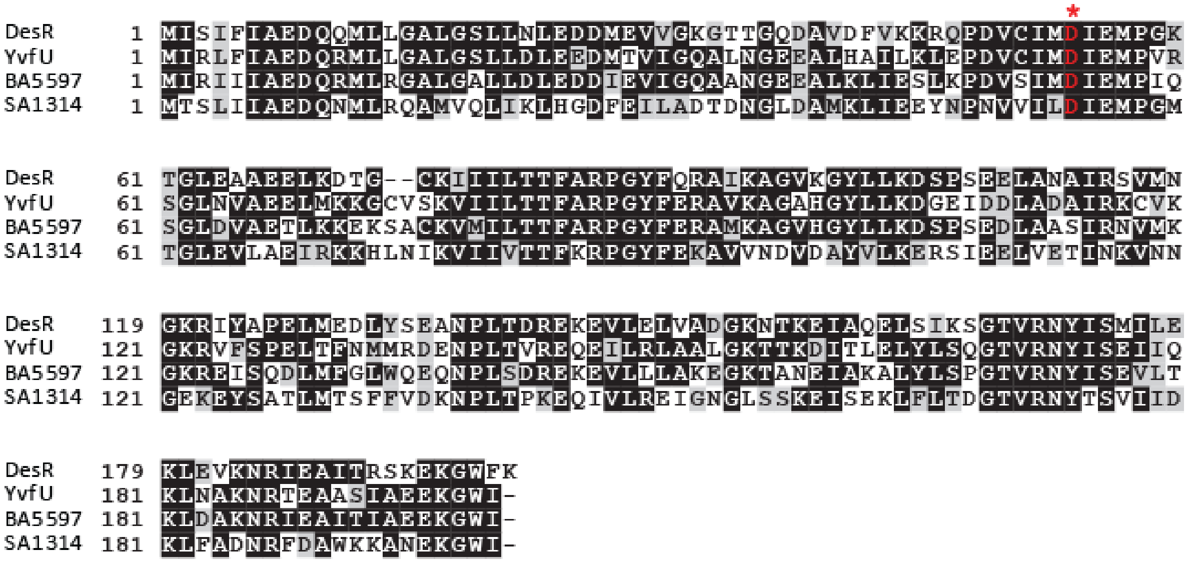

Fig. S1. Amino acid sequence alignment of RRs, DesR, YvfU, BA5597 and SA1314. Identical amino acids are shaded in black and similar ones are shaded in grey. The red asterisk highlights the phosphorylatable Asp in RR.

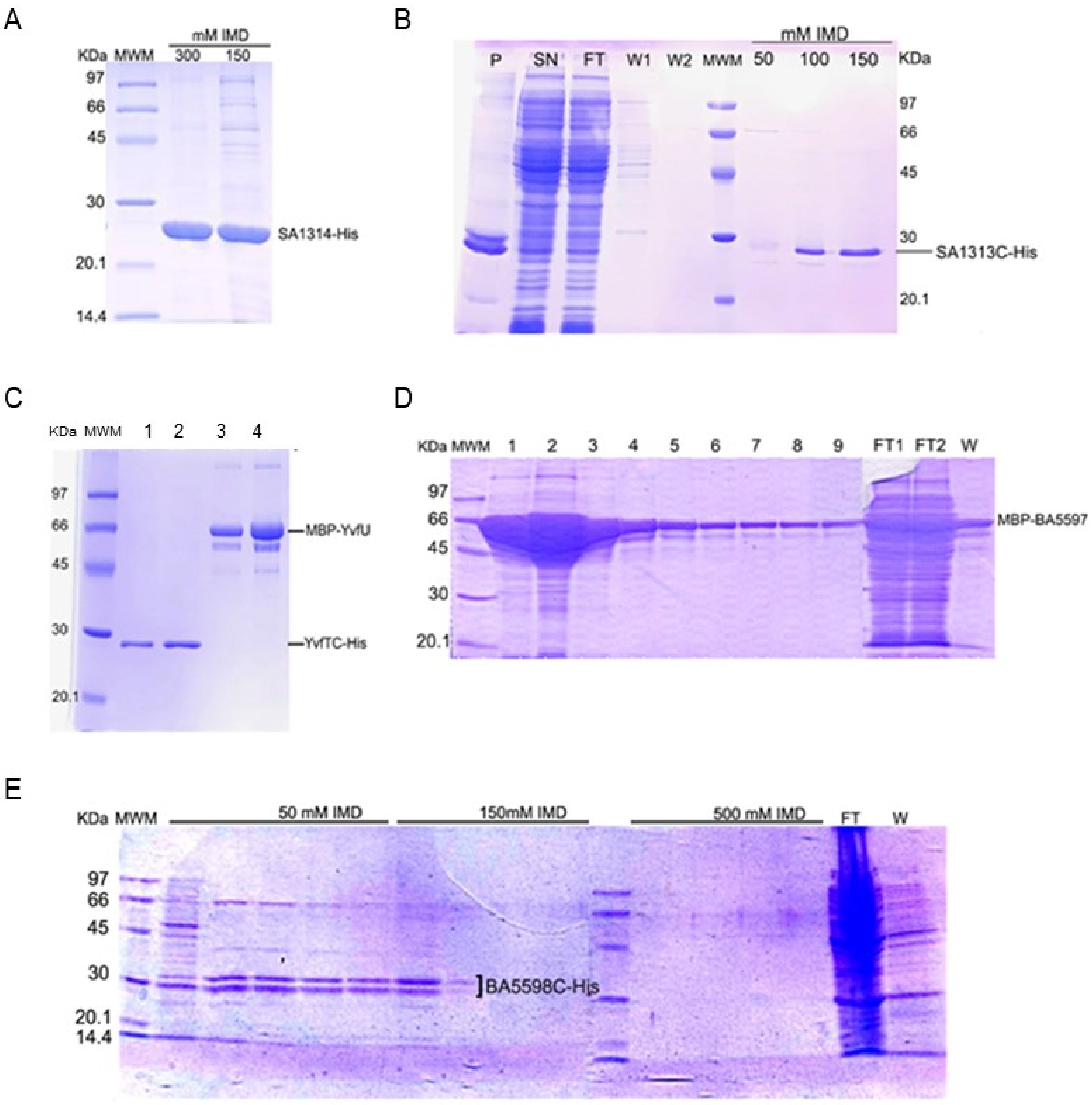

Fig. S2. Coomassie-stained SDS–PAGE of affinity-chromatography purified recombinant proteins. (A) Dialyzed samples of SA1314-His eluted with 300 and 150 mM imidazole, respectively. **(B)** SA1313C-His purification steps: P: pellet, SN: supernatant, FT: Flow through, W1 and W2: fractions of washing steps, and 50, 100 or 150 mM imidazole elution fractions. **(C)** Dialyzed samples of YvfTC-His and MBP-YvfU eluted with 150 mM imidazole or 10 mM maltose, respectively. 1 and 3: 10 µl aliquots; 2 and 4: 20 µl aliquots. **(D)** Purification steps of MBP-BA5597: 1-9 eluted fractions with 10 mM of maltose. **(E)** Purification steps of BA5598C-His showing eluted fraction with 50, 150 and 500 mM of imidazole. In all cases the cleanest fractions were dialyzed and conserved at -80°C until use. MWM: molecular weight marker

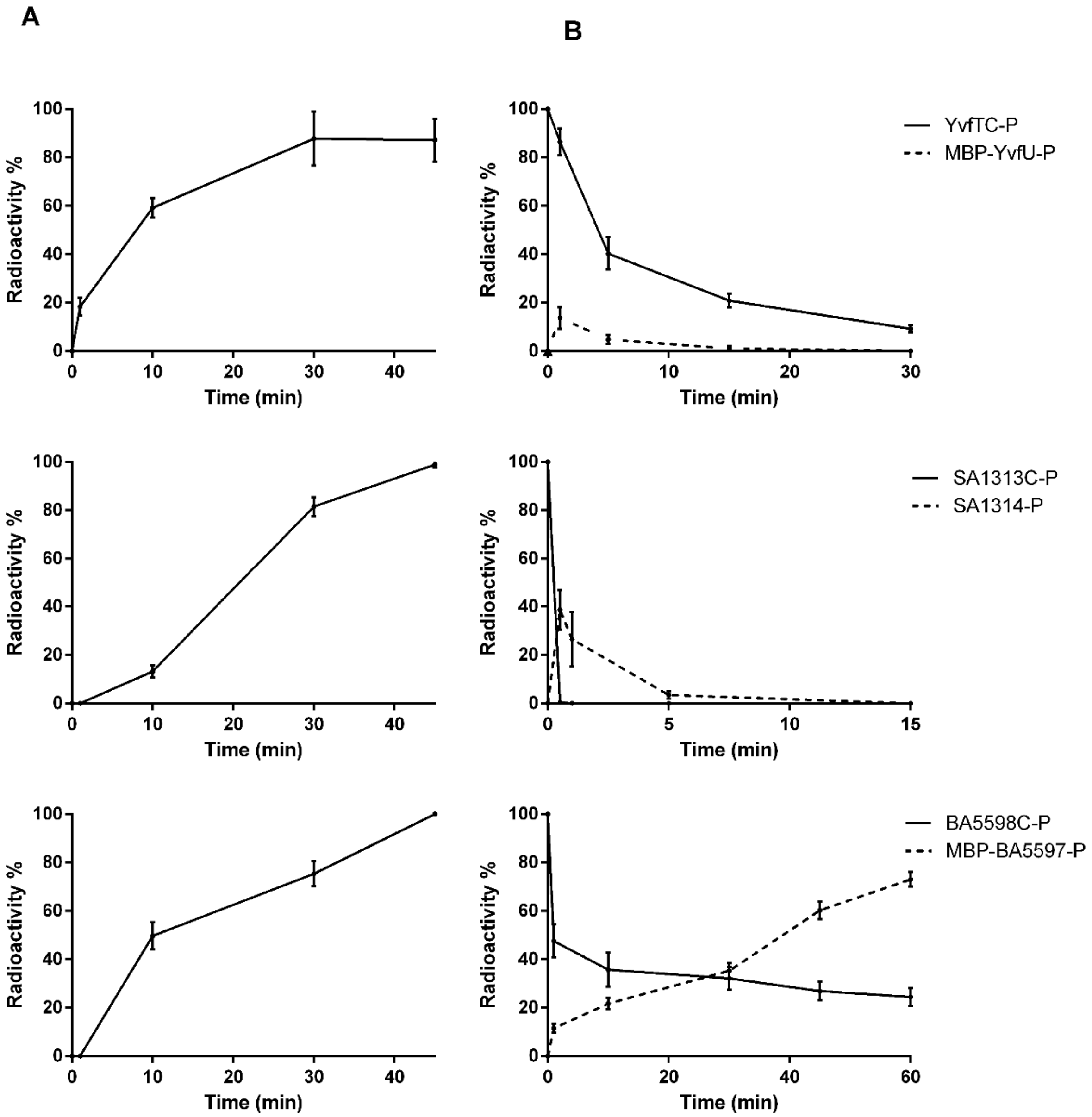

Fig. S3. Quantification of radiolabeled HKs and cognate RRs.

(A) Autokinase activity. The total amount of phosphorylated kinase present in each well was determined by densitometry and the maximum level obtained was considered 100%. Autophosphorylation labels were calculated from three independent experiments. Representative images are shown in Fig 3A.

(B) Phosphotransferase activity. The total amount of phosphorylated kinases and their cognate response regulators present in each well was determined by densitometry and the total label of HK-P at the initiation of the reaction time (0 min) was considered 100%. Results are the mean of three independent experiments, representative figures are shown in Fig. 3B. Radioactive labels were quantified by densitometry using Gel Pro Analyzer.

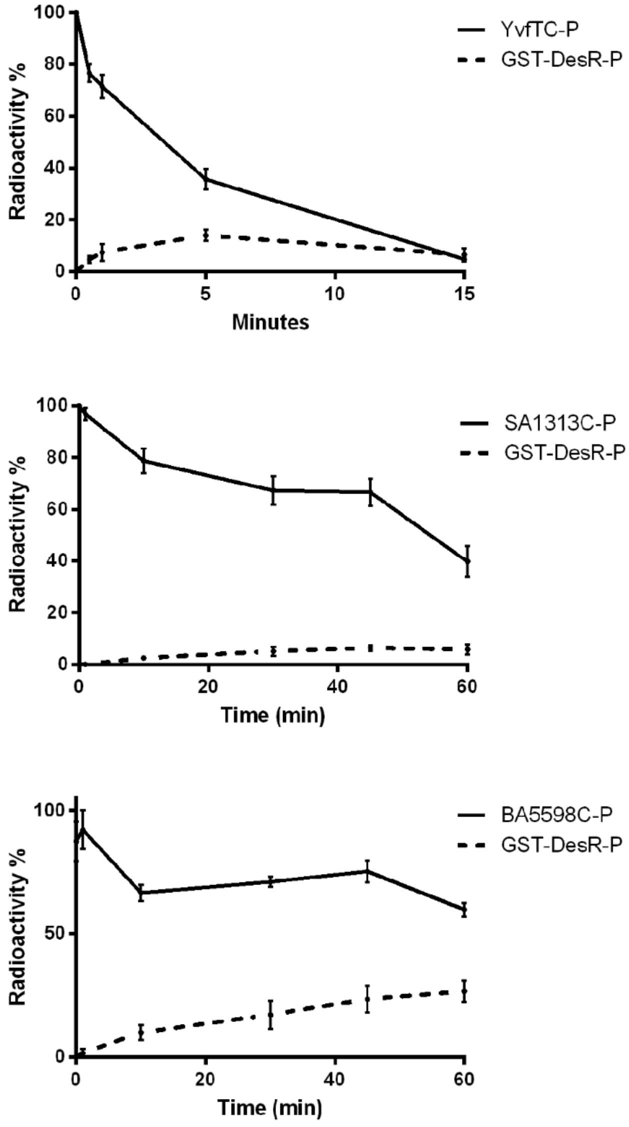

Fig. S4. Phosphotransfer from YvfTC, SA1313C and BA5598C to the non-cognate response regulator DesR.

The total amount of phosphorylated kinases and GST-DesR-P present in each well was determined by densitometry and the total label of HK-P at the initiation of the reaction time (0 min) was considered 100%. Results are the mean of three independent experiments, representative figures are shown in Fig. 4. Radioactive labels were quantified by densitometry using Gel Pro Analyzer.

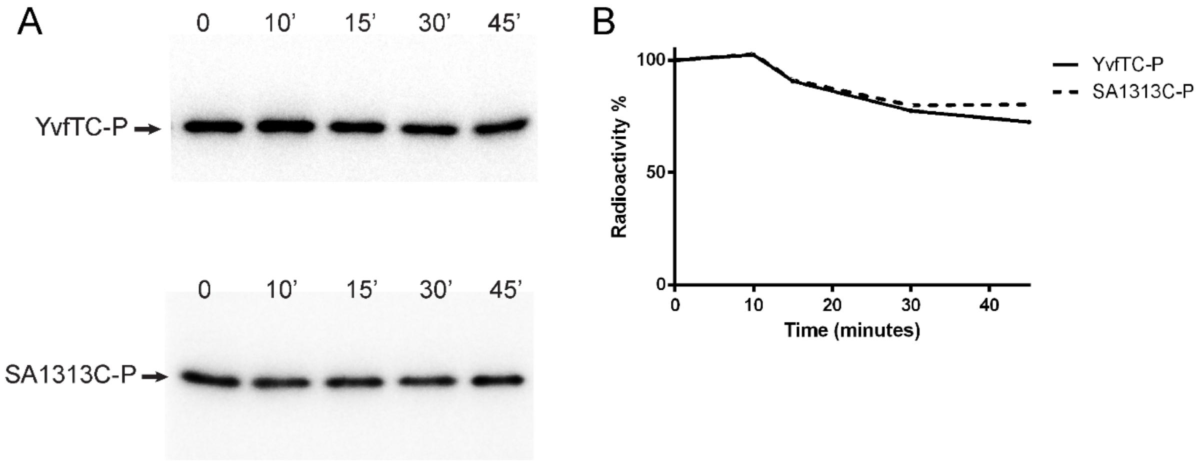

Fig. S5. (A) Phosphorylated HKs stability. The purified cytosolic domains were incubated in R buffer in the presence of [γ-^{32}P] ATP, at room temperature. Reaction mixtures were preincubated for 30 minutes and samples were taken at the indicated points. Reactions were stopped by the addition of 5X sample buffer. After SDS-PAGE gels were dried and exposed in Typhoon Fla 7000. (**B) Quantification of radiolabeled YvfTC-P and SA1313C-P**. Radioactive label was quantified by densitometry using Gel Pro Analyzer.

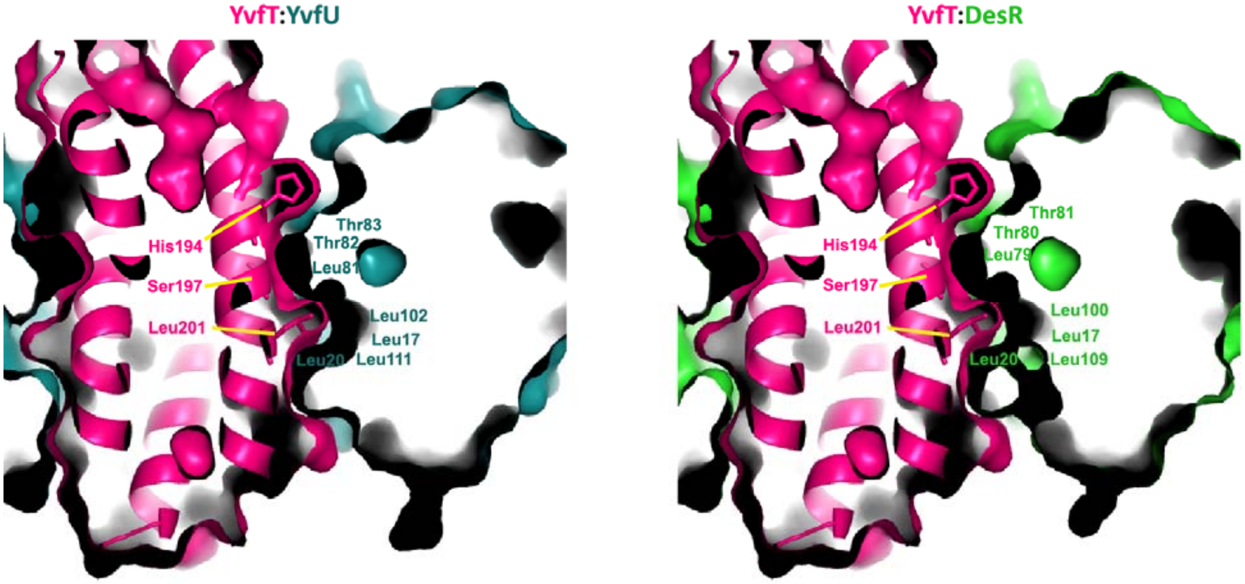

Fig. S6. Interaction surfaces of YvfT:YvfU and YvfT:DesR. Color reference indicating each protein is indicated in the top of the molecular models. YvfT is represented as cartoon and RRs as accesible surfaces. The aminoacids relevant for the interaction, based on crystallographic structures of DesK:DesR (Trajtenberg et al., 2016), are highlighted.

Bibliography

Trajtenberg, F., Imelio, J. A., Machado, M. R., Larrieux, N., Marti, M. A., Obal, G., et al. (2016). Regulation of signaling directionality revealed by 3D snapshots of a kinase:regulator complex in action. *Elife* 5. doi:10.7554/eLife.21422.
